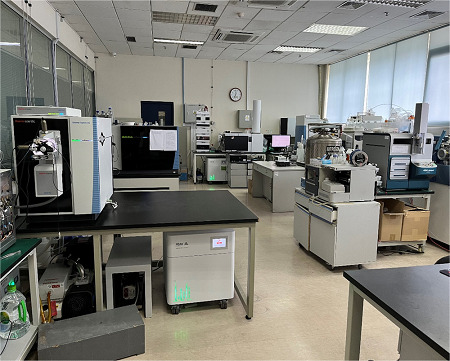# 基于成簇的规则间隔短回文重复序列的严重急性呼吸综合征冠状病毒2检测的最新进展

**DOI:** 10.3724/SP.J.1123.2022.08001

**Published:** 2022-09-08

**Authors:** Wen ZHOU, Kaiguang YANG, Lihua ZHANG, Zhen LIANG, Yukui ZHANG

**Affiliations:** 1.中国科学院大连化学物理研究所, 中国科学院分离分析化学重点实验室, 辽宁 大连 116023; 1. CAS Key Laboratory of Separation Science for Analytical Chemistry, Dalian Institute of Chemical Physics, Chinese Academy of Sciences, Dalian 116023, China; 2.中国科学院大学, 北京 100049; 2. University of Chinese Academy of Science, Beijing 100049, China

**Keywords:** 严重急性呼吸综合征冠状病毒2(SARS-CoV-2), 等温核酸扩增, 成簇的规则间隔短回文重复序列(CRISPR), SARS-CoV-2变体, severe acute respiratory syndrome coronavirus 2 (SARS-CoV-2), isothermal nucleic acid amplification, clustered regularly interspaced short palindromic repeats (CRISPR), SARS-CoV-2 variants

## Abstract

严重急性呼吸综合征冠状病毒2(SARS-CoV-2)导致的新冠肺炎(COVID-19)迅速蔓延全球,给全球公共卫生系统带来了挑战。由于逆转录-定量聚合酶链反应(RT-qPCR)和抗原测试的普遍适用性和灵敏度较差,并且具有不同突变的SARS-CoV-2变体持续的出现,给疫情防控带来了更大的挑战,因此,高灵敏度、无需设备并且能够区分SARS-CoV-2变体的诊断方法亟须发展。基于成簇的规则间隔短回文重复序列(CRISPR)的诊断对设备要求低,具有可编程性、灵敏性和易用性,已经发展出多种核酸检测工具用于传染病的诊断,其在临床上具有巨大的应用潜力。文章聚焦于近期发表的基于CRISPR实现SARS-CoV-2检测和变体区分的最新技术,总结其特点并对其发展进行了展望。

## Abstract (en)

The rapid global spread of coronavirus disease 2019 (COVID-19), caused by the severe acute respiratory syndrome coronavirus 2 (SARS-CoV-2), has introduced various challenges in global public health systems. The poor applicability and sensitivity of the reverse transcription-quantitative polymerase chain reaction (RT-qPCR) and antigen-based tests, as well as the persistent emergence of SARS-CoV-2 variants with different mutations hinder satisfactory epidemic prevention and control. Therefore, there is an urgent need for diagnostic technologies capable of distinguishing SARS-CoV-2 variants with high sensitivity and low (or no) equipment dependence. Diagnosis based on clustered regularly interspaced short palindromic repeats (CRISPR) has low equipment requirements and is programmable, sensitive, and easy to use. Various nucleic acid detection tools with great clinical potential have been developed for the diagnosis of infectious diseases. Therefore, this review focuses on the reported state-of-the-art CRISPR diagnostic technologies developed for the detection and differentiation of SARS-CoV-2 variants, summarizes their characteristics and provides an outlook for their development.

2019年12月底爆发的新冠肺炎(coronavirus disease 2019, COVID-19)迅速蔓延,截至2022年7月24日,全球累计确诊病例已经超过5.73亿,并且仍然呈上升趋势。国际病毒分类委员会将引发COVID-19的病毒命名为严重急性呼吸综合征冠状病毒2(severe acute respiratory syndrome coronavirus 2, SARS-CoV-2),它是一种单股正链RNA病毒。病毒动力学模型表明,高频次检测对于有效识别和隔离携带者以及遏制大流行至关重要^[1]^,因此,快速、高通量、高灵敏、高准确、低成本的SARS-CoV-2检测技术亟待发展。

SARS-CoV-2诊断的金标准是逆转录-定量聚合酶链反应(reverse transcriptase quantitative polymerase chain reaction, RT-qPCR),它具有高特异性和高灵敏度,但它需要特殊的设备和专业技术人员,且试剂盒短缺和巨大的检测需求导致样品应答时间长^[2,3]^。抗原捕获测试和具有可视读数的等温核酸诊断是在集中式实验室之外进行SARS-CoV-2测试的替代方法。抗原捕获测试快速且用户友好,但其较低的灵敏度可能导致假阴性结果^[4]^,它仅适用于高病毒滴度的受感染个体;等温核酸扩增方法,如环介导等温扩增(loop-mediated isothermal amplification, LAMP)和重组酶聚合酶扩增(recombinase polymerase amplification, RPA)比抗原捕获测试更灵敏^[5,6]^,但这些设备对于单次使用来说过于昂贵。因此,开发高灵敏度和无需设备的诊断方法是应对新冠肺炎疫情的关键。

成簇的规则间隔短回文重复序列(clustered regularly interspaced short palindromic repeats, CRISPR)/CRISPR相关(CRISPR-associated, Cas)系统在适应性细菌免疫中被首次发现^[7]^。基于CRISPR的诊断(CRISPR-Dx)对设备要求低,具有可编程性、灵敏性和易用性,是用于SARS-CoV-2检测的有前景的技术。CRISPR-Dx技术通常将等温核酸扩增方法与Cas12或Cas13结合。当CRISPR RNA(crRNA)与核酸特异性靶向结合后,Cas12或Cas13被激活,切割报告分子,释放荧光团,通过荧光信号检测病毒^[8,9]^。目前,两项CRISPR-Dx技术(Sherlock CRISPR SARS-CoV-2 Kit和SARS-CoV-2 RNA DETECTR ASSAY)已获得食品药品监督管理局(FDA)的紧急授权,可用于SARS-CoV-2的检测,但由于检测结果需要结合临床诊断共同判断,仅限于经批准的实验室使用。

SARS-CoV-2的进化导致具有多组突变的病毒变体的出现和持续传播,这些突变会增加传染性或减少抗体的中和作用,使病毒更难以控制。到目前为止,只有少数CRISPR-Dx被开发用于识别SARS-CoV-2变体中存在的核苷酸取代,但它们都不适用于现场检测^[10,11]^。因此,基于CRISPR开发高灵敏度、用户友好、无设备要求、快速、低廉的SARS-CoV-2检测技术,并实现SARS-CoV-2变体区分检测,对于应对COVID-19挑战是非常关键的。本文介绍近期发表的基于CRISPR实现SARS-CoV-2检测和变体区分的最新技术,对其进行评述。

## 1 基于核酸多重评估的组合阵列反应平台的CRISPR-Dx检测技术

大多数CRISPR诊断对于每个样品检测1~3个目标物,为了同时检测多种样品和多重病原体,Sabeti等^[12,13]^将基于CRISPR的核酸检测与微孔阵列系统集成,开发了核酸多重评估的组合阵列反应平台(combinatorial arrayed reactions for multiplexed evaluation of nucleic acids, CARMEN)。CARMEN利用核酸诊断的计算设计工具ADAPT (activity-informed design with all-inclusive patrolling of targets)设计crRNA,其能够在靶物种内提供高覆盖率,并且对其他物种具有高选择性^[14]^;输入的是PCR或RPA扩增的样品以及包含Cas13、序列特异性crRNA和报告分子的检测混合物。

每个扩增的样品和检测混合物与独特的荧光色码结合,随后乳化产生1 nL的液滴;来自所有样品和检测混合物的液滴装载到由聚二甲基硅氧烷模制的微孔阵列芯片中,芯片中的每个微孔随机容纳两个液滴,在电场中每个微孔的液滴对合并,同时开始检测反应,通过荧光显微镜监测反应(如图1)。

**图 1:**
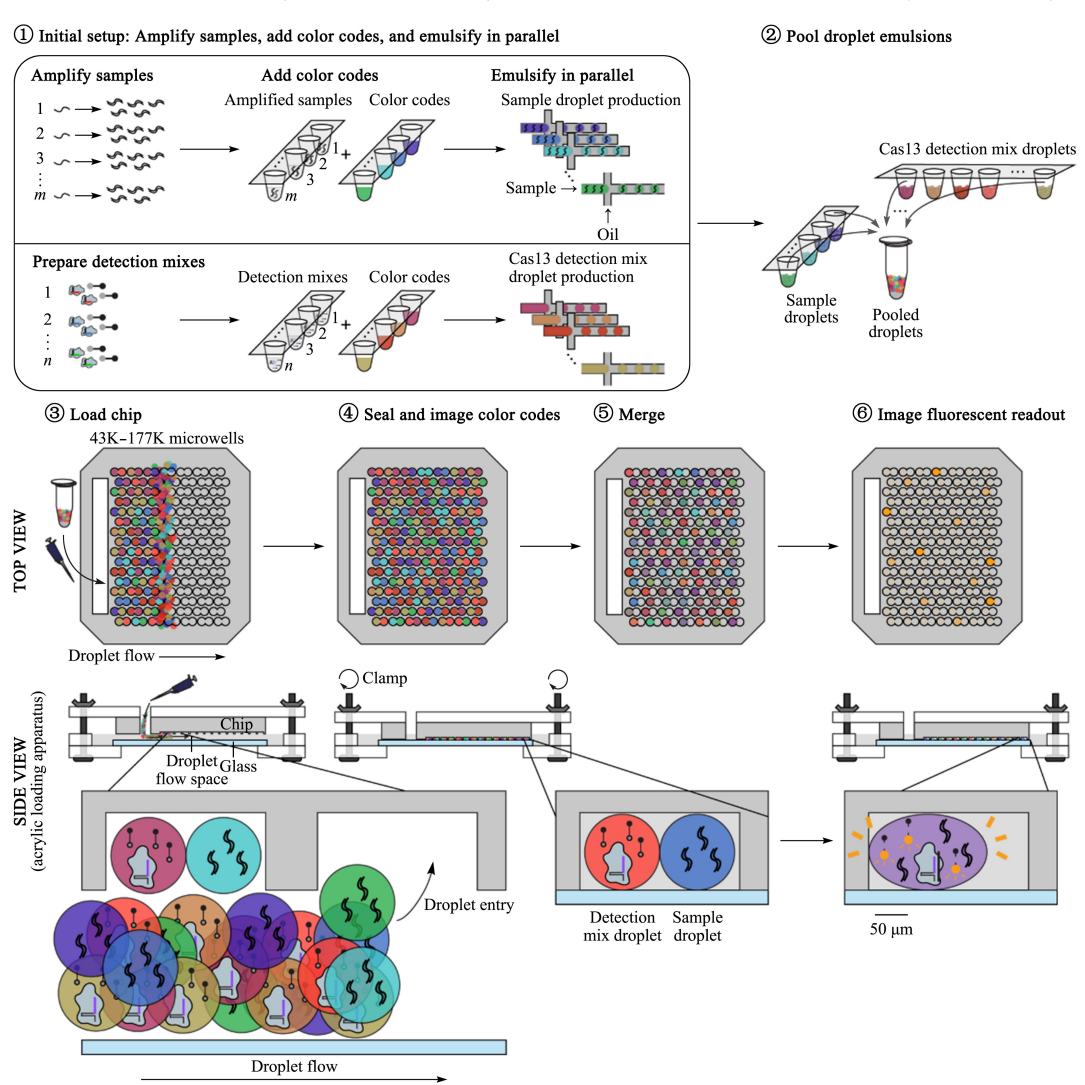
CARMEN中芯片功能详细原理图^[13]^

构建的CARMEN方法能够同时检测169种病毒,它实现了基于CRISPR的大规模诊断,其固有的多路复用和通量能力使其具有规模实用性,并且将每次测试的试剂成本降低为原来的1/300以下。CARMEN的灵活性可以允许添加新的扩增引物或crRNA以促进对SARS-CoV-2等新发现病原体序列的检测。

对于疫情,理想的诊断方法是同时具有处理上百份样本、检测多种病毒、区分病毒变体并量化病毒载量的能力^[15,16]^。第一代CARMEN(CARMEN v.1)虽然能够实现大规模诊断,但是它需要使用定制的成像芯片,且检测时间长达8~10 h^[13]^。为了满足快速检测多种病毒和变体的高通量临床检测需求,以CARMEN v.1为基础,结合商业的Fluidigm微流控技术,Sabeti等^[17]^继续构建出核酸多重评估的微流控组合阵列反应平台(microfluidic combinatorial arrayed reactions for multiplexed evaluation of nucleic acids, mCARMEN),它是迄今唯一的1种将监测功能结合到单一技术平台的诊断工具,能够在一天内测试数百份样本中的多种呼吸道病毒和变体,同时还能够量化病毒基因组拷贝数。

mCARMEN通过使用集成流路芯片(integrated fluidic circuit, IFC),消除了CARMEN v.1的颜色编码和滴状化需求;在IFC中混合大量的样品,避免了CARMEN v.1通过扩散进行液滴混合;通过利用Fluidigm微流控技术,克服了CARMEN v.1对定制显微镜和芯片的需求(如图2)。开发的mCARMEN呼吸道病毒检测板能够测试多达21种病毒。为了实现临床应用,对mCARMEN工作流程进行优化(如图3),通过实施自动化RNA提取,使用具有1个引物库的单步RNA-DNA扩增,并减少检测读数的持续时间,将人工劳动和处理时间减少到低于5 h。

**图 2:**
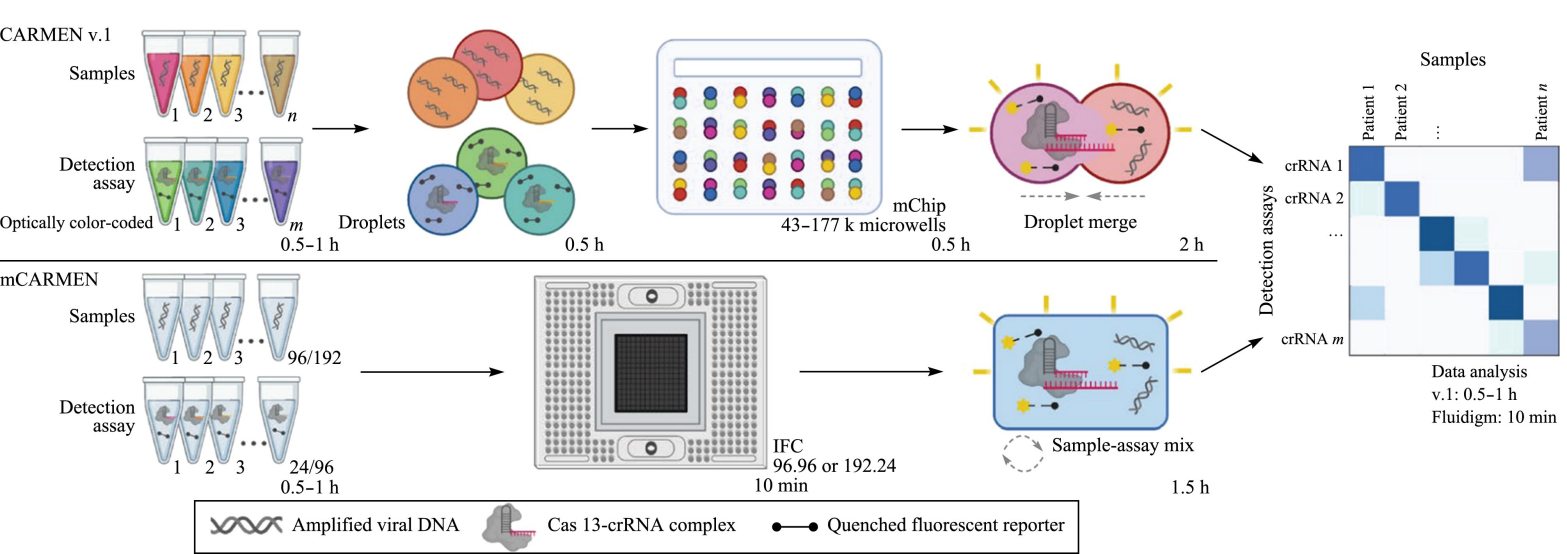
CARMEN v.1和mCARMEN工作流程示意图^[17]^

**图 3:**
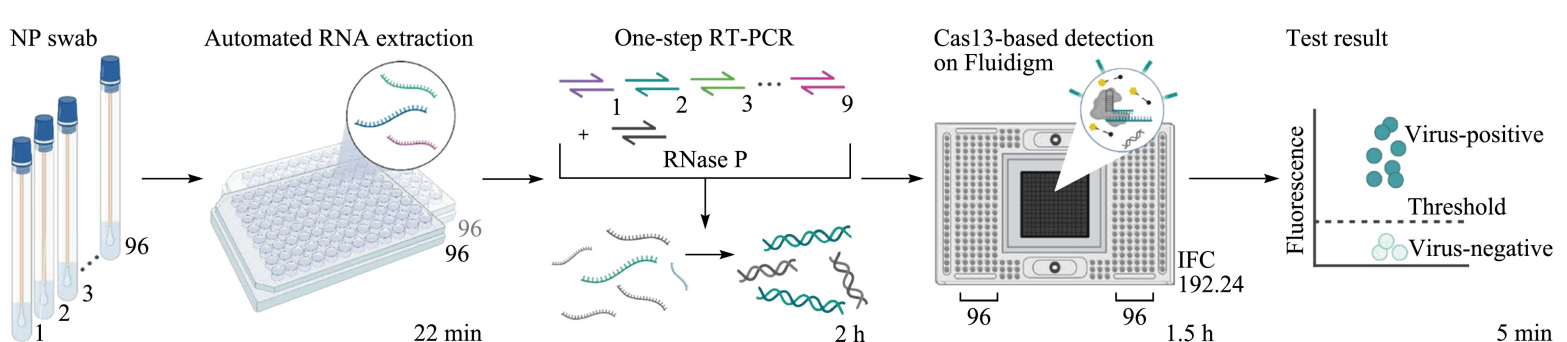
简化的mCARMEN工作流程示意图^[17]^

mCARMEN进一步开发呼吸道病毒面板(respiratory virus panel, RVP)来检测9种最具临床相关性的病毒(SARS-CoV-2、HCoV-HKU1、HCoV-OC43、HCoV-NL63、FLUAV、FLUBV、HPIV-3、HRSV和HMPV)。为了比较RT-qPCR和mCARMEN RVP的灵敏度,测试多次冻融循环不同浓度SARS-CoV-2样品(100、1000、10000拷贝数/mL)对检测重复性的影响,发现任何浓度下mCARMEN RVP的灵敏度不受冻融循环的影响;而RT-qPCR在最低浓度样本时受到冻融循环的负面影响,100拷贝数/mL的8个重复样本并不能被全部检出。说明mCARMEN RVP对于低病毒载量的样品检测效果更好,灵敏度更高,不受冻融循环的影响。

将mCARMEN RVP用于来自马萨诸塞州总医院的166份临床样本以及150份人工病毒样本的检测,所有的RVP病毒靶标均具有100%的阴性符合率;除人类偏肺病毒(human metapneumovirus, HMPV)外,所有的靶标均具有大于95%的阳性符合率,超过了FDA设定的最低临床性能标准。在137份临床阳性结果中,mCARMEN正确检测了95%(130份)的病毒核酸;对于150份人工病毒样本,mCARMEN正确识别了99%(148份)。

CARMEN v.1不能对样本中病毒基因组拷贝数进行定量评估,而确定患者体内的病毒总量对于评估感染阶段、传播风险和最有效的治疗方案非常重要^[15,16]^。为了使mCARMEN能够用于定量,利用具有不同反应动力学和酶活性的多个Cas相关蛋白(Cas12和灵敏度更高的Cas13分别捕获标准曲线上高拷贝数样本和低拷贝数样本的动力学曲线),以及Fluidigm Biomark检测的3个荧光通道,绘制荧光强度达到50%的时间和浓度关系的标准曲线。因此,联合使用Cas12和Cas13,可以对患者样本中跨越1~10^6^拷贝数/μL的SARS-CoV-2和甲型流感病毒进行定量。

目前,变异谱系分类仅由下一代测序技术(next-generation sequencing, NGS)进行评估,临床诊断不能很好地识别SARS-CoV-2变体中携带的突变(单核苷酸多态性,插入或缺失),因此用于全面检测26种SARS-CoV-2刺突基因突变的诊断和监测平台具有重要的现实意义。mCARMEN继续开发出了变体识别面板(variant identification panel, VIP),能够识别和区分6种SARS-CoV-2变体(Alpha、Beta、Gamma、Delta、Epsilon和Omicron),它在刺突基因的保守区域内有两个不重叠的引物对,用于扩增全长序列,利用突变设计的26个crRNA对允许追踪现有的变体并识别新出现的变体。

将mCARMEN VIP技术用于马萨诸塞州收集的1557份样本,通过将NGS确定的谱系结果与mCARMEN VIP谱系结果对比,发现存在99.5%的一致性(1549份),但NGS技术的检测周期比mCARMEN VIP长约4~7天;此外,NGS的每个样本成本比mCARMEN VIP高5~10倍。

## 2 基于特定高灵敏度酶解报告系统的CRISPR-Dx检测技术

LAMP能够以最低的设备要求进行高通量测试,但通常需要纯化的样品才能获得高灵敏度;改进的RPA能够用于未提取样品的测试,并具有更高的灵敏度,但仅与基于横向流的视觉读数兼容,这对于大批量样品测试是不可取的^[18]^。Abbott公司的检测仪ID NOW COVID-19测试使用未提取样品进行等温扩增,可在5~13 min内报告结果,但该技术需要昂贵的设备,且通量较低^[19,20]^。因此,等温扩增方法仍然需要技术进步,以便在实验室外以低成本和高通量进行测试。

基于CRISPR的诊断方法SHERLOCK(specific high sensitivity enzymatic reporter unlocking, SHERLOCK)^[21]^从提取核酸开始,包括两个独立的步骤:(1)等温RPA;(2)T7转录和Cas13介导的单链RNA报告分子的侧链切割。目前基于SHERLOCK的诊断与热处理未提取样品以灭活核酸酶(heating unextracted diagnostic samples to obliterate nucleases, HUDSON)的技术^[22]^兼容,使用热和化学还原灭活核酸酶并溶解病毒粒子,消除了对核酸提取的需要。这些方法将病毒检测设备和实验室基础设施的需求减少到只需一个加热元件。然而,它们的扩增产物需要在试管之间转移,这会增加污染和用户错误的风险。

为了解决当前核酸诊断的局限性,Sabeti等^[9]^建立了简化突出显示感染以应对流行病的方法(streamlined highlighting of infections to navigate epidemics, SHINE),可以从未提取的样本中检测SARS-CoV-2 RNA。SHINE是一种基于SHERLOCK^[21]^的SARS-CoV-2分析方法,具有以下优点:(1)将基于RPA的扩增和基于Cas13的检测合并为一个步骤,减少了用户操作和分析时间;(2)为了消除对纯化核酸的需求并减少总运行时间,通过添加核糖核酸酶抑制剂,将HUDSON孵育时间从30 min减少到10 min,以快速灭活样品中的病毒;(3)为了避免横向流读数时打开含有扩增产物的试管带来样品污染风险,结合了一个管内荧光读数器;(4)为了减少用户在解读管内读数结果时的偏差,通过配套的智能手机应用程序以自动化的方式解释荧光读数(如图4)。

**图 4:**
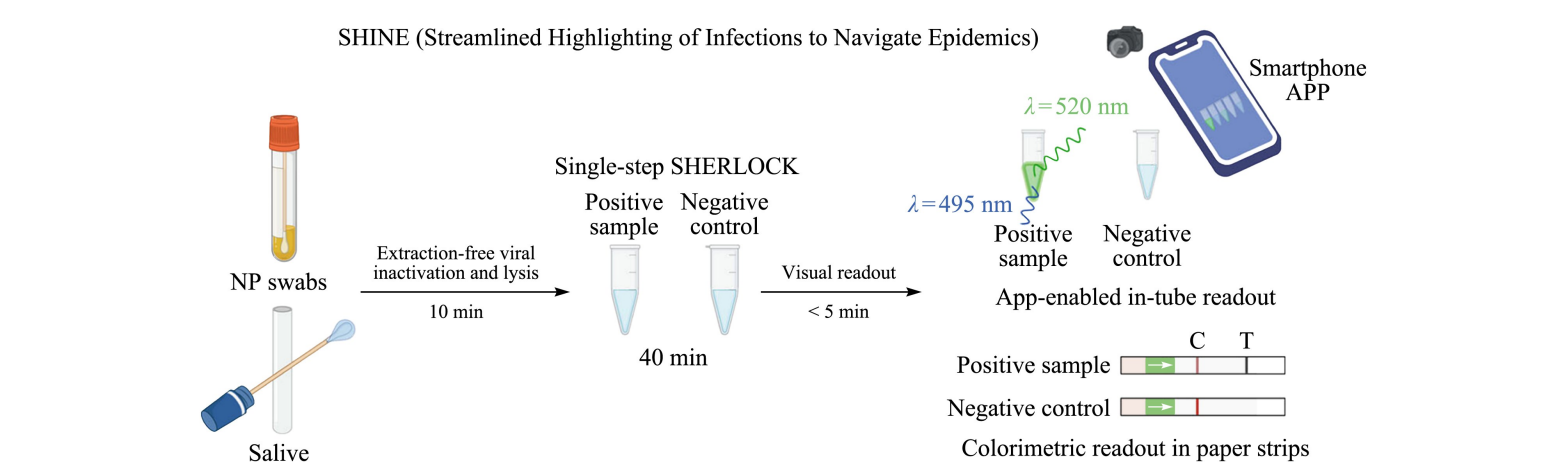
SHINE示意图^[9]^

使用样品应答时间50 min的SHINE测试了50个未提取的鼻、咽拭子样本,与RT-qPCR相比,灵敏度为90%(27/30, SARS-CoV-2阳性样本),并且具有100%的特异性。由于SHINE结合了用户友好、制备方法简单、灵敏度高、速度快的特点,其特别适合于社区监控。

第一代SHINE(SHINE v.1)^[9]^是一种不需要提取核酸或定制设备的诊断分析,然而SHINE v.1涉及多个加热步骤,并且需要低温储存和训练有素的人员手动制备试剂混合物。在此基础上,Sabeti等^[23]^继续开发的第二代SHINE(SHINE v.2)结合了室温样品处理和冻干测试试剂,从而消除对冷藏链的需求,大大方便了试剂运输和储存,降低了检测的整体复杂性。除此之外,SHINE v.1检测采用SARS-CoV-2开放阅读框1a(open reading frame 1a, ORF1a),与ORF1a相比,刺突基因检测速度快1~2倍,灵敏度高10倍。考虑到其增加的灵敏度和有利的动力学,SHINE v.2选择刺突基因检测用于进一步开发。

病毒裂解缓冲液(FastAmp^®^ Viral and Cell Solution)^[18]^能够在环境温度下发挥作用,SHINE v.2通过将含5% RNA酶抑制剂的FastAmp裂解试剂加入10%(鼻、咽拭子样本占通用运输介质的体积分数)的样品中处理5 min,在环境温度下快速、无设备地有效灭活核酸酶和SARS-CoV-2;通过在原始SHINE缓冲液中添加稳定剂蔗糖和填充剂甘露醇,去除不稳定成分聚乙二醇和氯化钾,增加冻干后SHINE缓冲液的活性,使其仍然能够保持冻干前SHINE缓冲液的检出限。SHINE v.2大大减少了用户的实际操作时间和液体处理步骤,从SHINE v.1的45 min和20个移液步骤减少到不足10 min和5个用户操作(如图5)。

**图 5:**
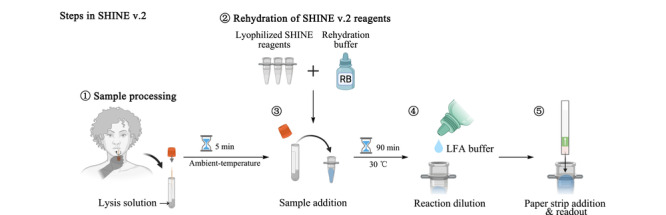
SHINE v.2工作流程示意图^[23]^

以RT-qPCR为基准,利用SHINE v.2对SARS-CoV-2阳性和阴性患者共72份鼻、咽拭子样本(病毒载量代表了一般人群,中值病毒载量约10^7^ 拷贝数/mL)进行测试,SHINE v.2能够以90.5%的灵敏度(38/42, 阳性样本)和100%的特异性检测未提取的鼻、咽拭子中的SARS-CoV-2 RNA。随后将SHINE v.2与两种广泛使用的FDA紧急授权的抗原捕获测试(Abbott的BinaxNow新冠肺炎抗原自测^[24]^, Access Bio的CareStart新冠肺炎抗原检测^[25]^)进行比较。在另外一组96份鼻、咽拭子样本中(病毒载量分布比一般人群低50~100倍,中值病毒载量约1.9×10^5^拷贝数/mL以上), SHINE v.2比两种抗原捕获测试的灵敏度都高50倍。SHINE v.2擅长检测中等病毒载量的样本,可以识别抗原捕获测试可能遗漏的潜在感染个体,并且具有100%特异性,在33个RT-qPCR阴性样品中均无假阳性结果。

为了鉴别SARS-CoV-2的不同变体,SHINE v.2针对不同变体的标志设计了具有不同活性的原始RNA靶标和含有给定突变的衍生RNA靶标的crRNA组,并且通过ADAPT^[14]^帮助设计RPA引物,以检测区分5种变体(Alpha、Beta、Gamma、Delta、Omicron)。使用Omicron特异性的SHINE v.2,对12个未提取的鼻、咽拭子的临床样本进行测试,正确鉴定出所有Omicron阳性样本中142~145突变的存在,对照的Omicron阴性样本中没有检测到142~145突变。

基于Cas13a的SHERLOCK平台^[21]^, Pecori等^[26]^开发出通过优化SHERLOCK准确检测进化的SARS-CoV-2诊断测试方法(accurate detection of evolving SARS-CoV-2 through SHERLOCK optimization, ADESSO),直接从患者样本中对SARS-CoV-2及其变体进行高灵敏度检测。该方法在大约1 h内完成(如图6),且不需要RNA提取和任何特定设备,具有2.5拷贝数/μL SARS-CoV-2合成基因组的检测极限(接近RT-qPCR的极限)。使用的基于横向流的视觉读数是一种无需仪器的检测方法,在阴性样品中,RNA报告分子是完整的,并被链霉亲和素捕获,产生一条对照带;在阳性样品中,报告分子被切割,释放出含有荧光团的片段,并被金标记的抗体捕获,产生测试带。测试带和对照带之间的带强度比反映了Cas13的激活水平,条带强度比高于0.2的样品即为阳性样品。

**图 6:**
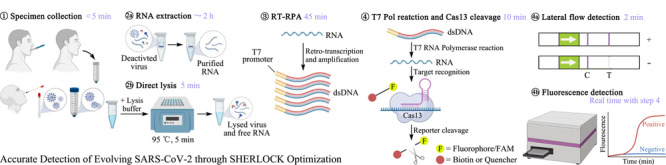
ADESSO在临床样本中检测SARS-CoV-2的实验工作流程图^[26]^

使用ADESSO测试共195份SARS-CoV-2阳性和阴性临床样本,将其与RT-qPCR和抗原测试进行比较。RT-qPCR和ADESSO通过提取的RNA进行分析比较;基于抗原的诊断测试(RIDA^®^QUICK SARS-CoV-2 Antigen)和ADESSO直接在未提取的样本上进行比较。在提取的RNA上,ADESSO和RT-qPCR具有相当的灵敏度和特异性(ADESSO和RT-qPCR灵敏度分别为96%和94%);在未提取的拭子样品上,ADESSO的灵敏度显著优于抗原测试(ADESSO和抗原测试的灵敏度分别为77%和46%)。结合之前数学模型结果:成功识别和隔离50%的受感染个体足以使感染曲线趋缓^[27]^。因此,ADESSO对于控制疫情有很大的帮助。

对标准ADESSO进行调整,以检测SARS-CoV-2不同变体(Alpha、Beta、Delta和Omicron)。针对每种变体的特异性突变,设计了crRNA来识别这些序列。在标准ADESSO的基础上,由于crRNA和靶标RNA之间少量的错配,Cas13反应时间由10 min调整至20 min;此外,还观察到交叉变异反应的信号略有增加,因此将条带强度比阈值从0.2增加到0.4,以避免变体鉴定中的假阳性。将每种变体特异性的ADESSO用于临床样本,能够成功鉴定其中的所有变体。

ADESSO的单次反应成本低于5 C=,这与抗原检测相当,但是灵敏度更高;与基于RT-qPCR的新冠肺炎诊断检测相比更低廉,并且能够实现更广泛和更高频次的检测。但是ADESSO是一种两步法的CRISPR-Dx技术,它处理步骤多,并且容易引入样品污染的风险。因此,未来研究有必要在保持高灵敏度的同时,探索“一锅法”ADESSO。

## 3 总结与展望

由于RT-qPCR和抗原测试的普遍适用性和灵敏度较差,仍然需要一种在灵敏度和特异性方面与RT-qPCR相当,但更快且不依赖于复杂仪器的替代检测技术。基于CRISPR的大规模诊断具有高通量、快速检测、用户友好、无需设备、低廉、高灵敏度和特异性的性质,这促使它成为公共卫生系统应对SARS-CoV-2的关键,能够实现常规、全面的监测。

基于CARMEN的CRISPR诊断技术,对病毒样本进行RNA的提取与扩增,能够同时实现多种样品、多重病毒的高通量检测,mCARMEN在CARMEN v.1的基础上,使用IFC消除颜色编码的需求,使用Fluidigm微流控技术克服定制芯片的需求,并联合使用Cas12和Cas13实现病毒拷贝数的量化,实现了SARS-CoV-2变体的区分。基于SHERLOCK的CRISPR诊断技术,直接分析灭活的病毒样本,无需RNA提取。SHINE v.1合并了基于RPA的扩增和基于Cas13的检测,通过配套的手机应用程序自动化解释荧光读数;SHINE v.2在此基础上结合了室温样品灭活处理和冻干测试试剂,以简化试剂运输和储存过程。并且检测灵敏度更高的刺突基因代替了SHINE v.1的ORF1a。

基于CRISPR的诊断技术具有很好的适应性,更换crRNA可以使CRISPR针对不同的病毒和变体,能够快速应对当前和未来即将爆发的其他疫情的诊断需求。未来基于CRISPR的诊断工具需要在不牺牲灵敏度或特异性的情况下,简化实验流程、提高用户可操作性、降低分析复杂性,并且具有低成本、快速检测的特点,能够实现传染病的高频次、高覆盖检测,加强公共卫生系统对传染病的控制与监测。

**作者团队简介**

中国科学院大连化学物理研究所生物分子高效分离与表征研究组(1810组)隶属于国家色谱研究分析中心、中国科学院分离分析化学重点实验室和中国科学院大连化学物理研究所生物技术部。自创立于1999年以来,研究组得到了迅速发展。在学术带头人和课题组组长的带领下,一直致力于蛋白质组高效分离与表征新技术新方法的研究,承担了多项国家级科研项目,并与国内外相关领域的知名学者建立了广泛的合作关系。课题组网站:http://www.proteomics.dicp.ac.cn/。

**人才队伍**

**学术带头人:** 张玉奎院士

**课题组组长:** 张丽华研究员

**职工及学生:** 研究员6人,项目研究员1人,副研究员4人,高级实验师3人,实验师2人,工程师1人,博士后及研究生40余人

**团队精神:** 做人做事做学问,求真求实求创新


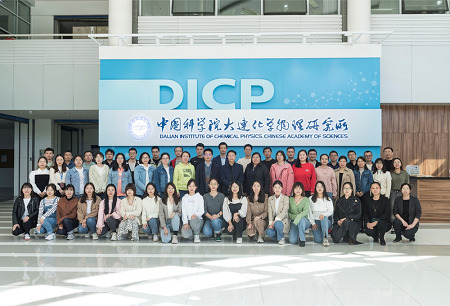


**科研项目及成果**

**科研项目:** 国家重大科学研究计划,国家基础研究计划,国家科技支撑计划,国家自然科学基金,中科院知识创新工程等

**科研成果:** 在*Nature Communication*, *Small*, *Advanced Materials*, *Analytical Chemistry*等期刊发表SCI论文650余篇,他引16000余次;申请发明专利280余项

**获奖情况:** 国家自然科学二等奖,辽宁省自然科学一等奖,辽宁省科技进步二等奖等

**研究领域**


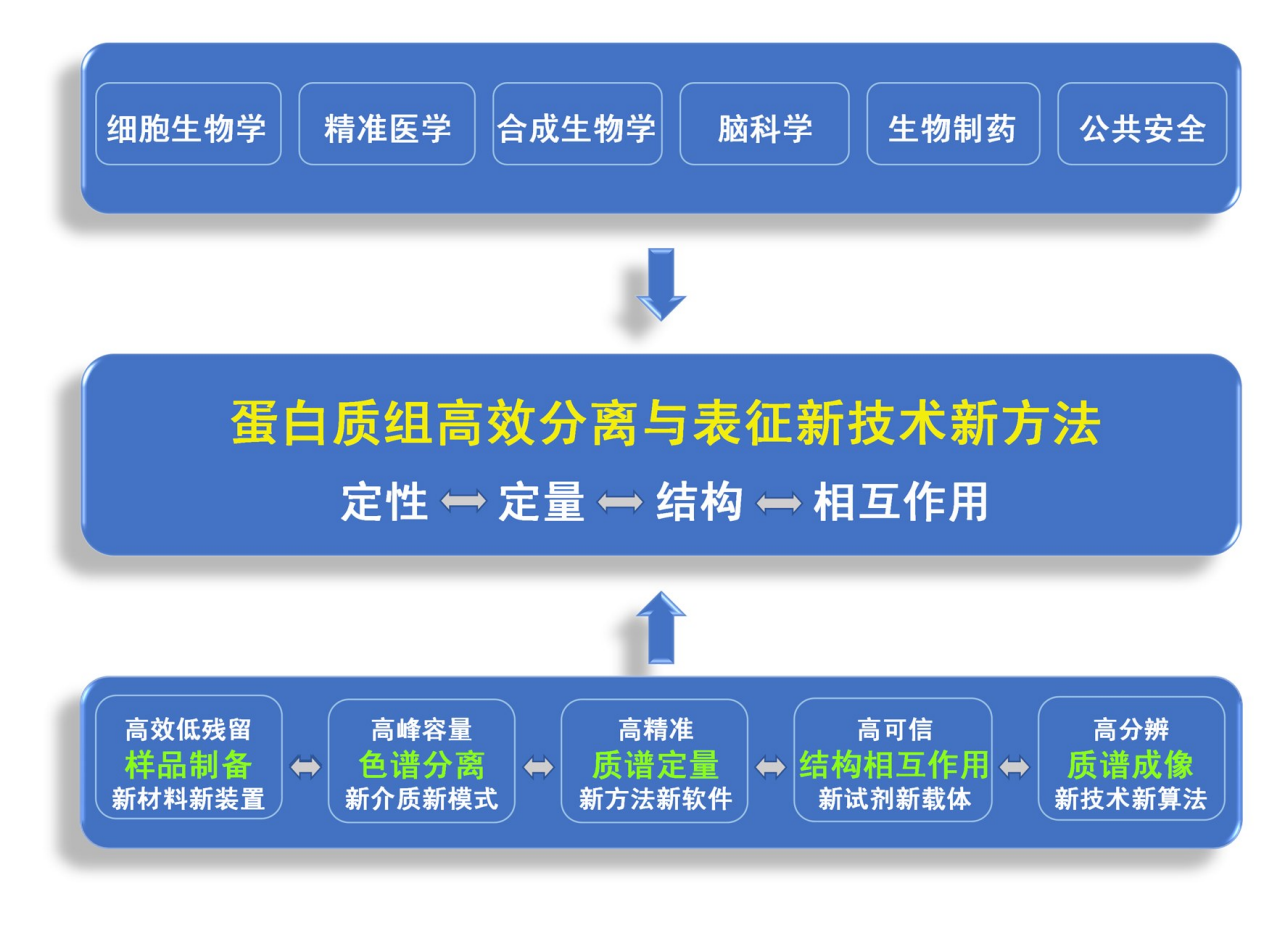


**仪器设备信息**

**实验室:** 质谱实验室,色谱实验室,细胞培养室等

**仪器设备:** Orbitrap Exploris 480高精度质谱仪,Orbitrap Fusion Lumos三合一高分辨质谱系统,Q-Exactive高分辨质谱仪,Orbitrap Velos离子阱质谱仪,Triple-TOF 5600+四极杆高分辨质谱仪,LTQ-ETD离子阱质谱仪,Ultra Flex Ⅲ MALDI-TOF-TOF质谱仪,1260 UHPLC-6540 QTOF MS联用分析系统,蛋白质分离纯化系统,UltiMate 3000微纳升多维液相色谱仪,1290超高压液相色谱仪,凝胶成像系统等